# CHOPER Filters Enable Rare Mutation Detection in Complex Mutagenesis Populations by Next-Generation Sequencing

**DOI:** 10.1371/journal.pone.0116877

**Published:** 2015-02-18

**Authors:** Faezeh Salehi, Roberta Baronio, Ryan Idrogo-Lam, Huy Vu, Linda V. Hall, Peter Kaiser, Richard H. Lathrop

**Affiliations:** 1 Department of Computer Science, University of California Irvine, Irvine, CA, 92697, United States of America; 2 Department of Biological Chemistry, University of California Irvine, Irvine, CA, 92697, United States of America; 3 Institute for Genomics and Bioinformatics, University of California Irvine, Irvine, CA, 92697, United States of America; 4 Chao Family Comprehensive Cancer Center, University of California Irvine, Irvine, CA, 92697, United States of America; The University of Hong Kong, HONG KONG

## Abstract

Next-generation sequencing (NGS) has revolutionized genetics and enabled the accurate identification of many genetic variants across many genomes. However, detection of biologically important low-frequency variants within genetically heterogeneous populations remains challenging, because they are difficult to distinguish from intrinsic NGS sequencing error rates. Approaches to overcome these limitations are essential to detect rare mutations in large cohorts, virus or microbial populations, mitochondria heteroplasmy, and other heterogeneous mixtures such as tumors. Modifications in library preparation can overcome some of these limitations, but are experimentally challenging and restricted to skilled biologists. This paper describes a novel quality filtering and base pruning pipeline, called Complex Heterogeneous Overlapped Paired-End Reads (CHOPER), designed to detect sequence variants in a complex population with high sequence similarity derived from All-Codon-Scanning (ACS) mutagenesis. A novel fast alignment algorithm, designed for the specified application, has *O(n)* time complexity. CHOPER was applied to a p53 cancer mutant reactivation study derived from ACS mutagenesis. Relative to error filtering based on Phred quality scores, CHOPER improved accuracy by about 13% while discarding only half as many bases. These results are a step toward extending the power of NGS to the analysis of genetically heterogeneous populations.

## Introduction

Next-generation sequencing (NGS) is a developing research area with an extensive growth of applications [1–3]. The high coverage achievable with NGS methods has enabled the detection of many low-frequency variants, including somatic mutations across the genome [1,4,5]. In these traditional applications of NGS, the cell population has a homogeneous genome, and so single nucleotide polymorphisms (SNPs) can be differentiated from sequencing errors by their rate of occurrence [4,6–8]. However, this strategy fails at detection of minor variations within genetically heterogeneous populations, because sequencing error rates associated with current NGS methods are difficult to distinguish from biologically important low-frequency variants. Approaches to overcome these limitations are essential for efficient detection of variants in large cohorts, rare mutations in virus or microbial populations, as well as description of mitochondria heteroplasmy and other heterogenic mixtures such as tumors [9–13]. Modifications in library preparation also can overcome these limitations, but are experimentally challenging and restricted to skilled biologists [14].

In this paper, we carried out a two-arm study that directly compared traditional sequencing against NGS on the task of heterogeneous mutation detection. The experimental target was a complex heterogeneous population with high sequence similarity that was derived from All-Codon Scanning (ACS) mutagenesis [15]. ACS is a mutagenesis method that generalizes traditional alanine scanning. ACS creates a defined gene library wherein each individual codon within a specific target region is changed simultaneously into all possible codons, while producing only a single codon change per mutagenesis product. Specifically, we searched for single amino acid changes that restore the activity of the tumor suppressor protein p53 carrying the cancer mutation M237I (mutation of methionine [ATG] to isoleucine [ATA] at p53 codon position 237). p53-M237I is a cancer mutation that is found frequently in human tumors; understanding its structure-function relation has considerable clinical relevance [16–18]. Occurrence frequency of individual mutations in heterogeneous ACS libraries is lower than the sequencing error rate associated with NGS, and previously this problem has precluded identification of these biologically meaningful variants. To overcome this limitation we developed a series of quality filtering and base pruning operations, called Complex Heterogeneous Overlapped Paired-End Reads (CHOPER) filtering, that together provide novel error filtering and mutation detection in the complex heterogeneous population derived from ACS mutagenesis [15]. A novel fast sequence alignment algorithm with *O(n)* time complexity was developed specifically for the CHOPER filtering approach.

Our experimental NGS method used complete overlapped paired-end reads of Illumina technology followed by computational error filtering. Relative to traditional sequencing, NGS provided a complete and informative picture of the mutational space and found every actively growing mutant present in the sequencing library. Our computational methods increased the average NGS accuracy of all p53 cancer mutant M237I codon positions from 74.51% to 99.73% at the expense of discarding only 21.28% of bases. When compared to NGS error filtering based on Phred quality scores alone, CHOPER filters reduced 13% more errors and discarded only half as many bases. Our novel *O(n)* sequence alignment method was 10 times faster than the Smith-Waterman algorithm for the specific problem addressed here, without sacrificing accuracy. The novel CHOPER active mutation detection approach reported here is a step toward extending the power of NGS to the analysis of important problems with genetically heterogeneous populations.

## Materials and Methods

### Preparation of ACS libraries

ACS libraries were generated as described in [15]. Briefly, ACS saturation mutagenesis was performed in the p53 region corresponding to amino acids 100 to 299, which encompasses the p53 core domain. The p53 cancer mutant M237I (mutation of methionine [ATG] to isoleucine [ATA] at p53 codon position 237) was used as the template. It was combined with every other possible single codon change through generation of the ACS library [15]. ACS library products were re-amplified from the mutagenesis mix by PCR. The PCR products were purified (QIAquick PCR purification kit, Qiagen, Germany) and used to generate the p53-expression plasmids *in vivo* using the gap-repair strategy in yeast cells [15,19]. Gap-repair resulted in a pool of yeast transformants with each cell containing one p53 expression plasmid, whereby each cell contained the p53-M237I cancer mutation plus exactly one other codon mutation introduced by ACS into the p53 core domain. In order to identify single-codon mutations that reactivate the p53 cancer mutant M237I, we used the p53-tester yeast strain RBy379 (1cUASp53::URA3 his3Δ200 a/alpha) for the gap-repair strategy [15,19,20]. In this strain the *URA3* gene is engineered to be under the transcriptional control of human p53. Therefore, growth of this yeast strain in a medium lacking uracil depends on active p53, allowing selection for intragenic second-site suppressor mutations that correct the defect caused by the cancer mutation M237I. In addition to the p53-dependent *URA3* selection marker, the plasmid reconstituted through *in vivo* gap-repair contains a *HIS3* nutritional marker. Cells require the intact *HIS3* gene for growth in medium lacking histidine. Growth in medium lacking histidine ensures the presence of the plasmid, but, importantly, does not require active p53.

### Identification of rescue mutations by traditional sequencing

The ACS library built with the p53 cancer mutation M237I was transformed into the p53-tester yeast strain RBy379 and transformants were plated onto agar plates lacking uracil. Only cells containing active p53 can form colonies on these plates. p53-containing plasmids were isolated from these colonies [21] and the p53 core domain from each colony was sequenced individually (Genewiz, San Diego, CA, USA).

### Identification of rescue mutations by Illumina sequencing

To develop the sequence data analysis strategies reported here we prepared three different samples for analysis by Illumina sequencing. These three samples are referred to as M237I, M237I_ACS, and M237I_RESCUE (see description of these three libraries in Table A in S1 File).

Sample M237I represents the unprocessed p53 core domain that contains the cancer mutation M237I but no other introduced mutations. This sample was prepared to analyze and control the baseline error rates associated with the procedure, as well as the intrinsic NGS sequencing error rates. The M237I core domain was amplified by PCR, without undergoing ACS, and transformed into the p53-tester yeast strain RBy379. Transformants were grown on agar plates lacking histidine to select for colonies containing the gap-repaired plasmid. Colonies were collected after 3 days of incubation at 30°C, pooled, inoculated in liquid medium lacking histidine, and cultured at 30°C until mid-log phase. Finally, plasmid DNA was extracted [21], the region corresponding to amino acid 50–334 was re-amplified by PCR, and the sample was prepared as described below for NGS. The M237I sample did not undergo any selective pressure other than requiring cells to contain the plasmid.

Sample M237I_ACS corresponds to ACS mutagenesis performed on the p53 core domain containing the cancer mutant M237I. The sample was processed as above by growth in media lacking histidine. Therefore, no selective pressure for p53 activity was applied. This sample controls for, and allows analysis of, the diversity of the ACS library.

Sample M237I_RESCUE is identical to M237I_ACS except that transformants were selected for active p53 by culturing the cells in media lacking uracil, thus requiring active p53 for growth. This sample was processed as above but transformants were directly plated onto plates lacking uracil and incubated at 37°C (the optimal temperature for human p53 activity). The resulting colonies were collected, cultured as a single pool in liquid medium lacking uracil until mid-log phase was reached, and processed as above. Importantly, the M237I_RESCUE sample was the only sample that was exposed to selective pressure requiring p53 activity.

**Illumina Sequencing.** PCR fragments amplifying the region encoding amino acids 50–334 for the three samples were prepared for Illumina sequencing as follows. Fragments were sheared independently in the Covaris DNA/RNA shearing NGS (Covaris Inc., Woburn, MA, USA) for 690 s. These conditions were determined to produce sheared products in the range of 130–150 bp. This small fragment size was critical to achieve maximal overlap of the paired-end reads. 10 ng of each sheared sample was barcoded using NEXTflex ChIP-Seq Library Systems Multiplex from Bioo Scientific (NEXTflex ChIP-Seq Barcodes, Bioo Scientific Corporation, Austin, TX, USA), according to manufacturer’s instructions. Linker and barcode addition increased the average fragment size to about 250 bp. Accordingly, libraries were size selected at 250 bp, followed by absolute quantification using KAPA qPCR (Kapa Biosystems, MA, USA), according to the manufacturer’s instructions. Finally, the three barcoded libraries were combined. A PhiX control library (Illumina, CA, USA) was added to the samples because the high sequence similarity of samples M237I, M237I_ACS, and M237I_RESCUE otherwise would trigger an error message through the automatic library quality control system of the HiSeq instrument. The NGS experiment was performed on an Illumina HiSeq 2500 sequencer (Illumina, CA, USA), using the program Rapid Run 150 Paired End (PE). Paired-end sequencing was chosen for efficient detection of sequencing errors because it provided two independent reads from the same DNA molecule. To ensure maximum overlap, it was important to achieve a short library fragment size during the DNA shearing step.

### Validation of rescue mutations

Cell growth assays under conditions depending on p53 activity were performed to validate experimentally the reactivation of p53 cancer mutant M237I by the rescue mutations that were detected through NGS sequencing. We used the p53-tester yeast strain RBy379 containing either wild-type p53, the p53 cancer mutant M237I, or the cancer mutant M237I combined with each of the identified rescue mutations. Each stock culture was grown overnight in small flasks at 30°C with orbital shaking at 125 RPM in medium lacking histidine to ensure the presence of the plasmid. Cells were washed in the culture medium and 150 μL of each culture, containing 1 million cells, was plated into Corning 96-well flat-bottomed clear plates. Cell growth was assessed by light scatter measurements made at 600 nm in a Tecan GENios Plus (Serial number: 502000004; Firmware: V 6.02 16_06_2004 Genios; XFLUOR4 Version: V 4.51; Tecan Group Ltd, Switzerland). Optical density was determined every 4 hours with continuous orbital shaking mode employed for a total of 32 hours. Temperature control was enabled at 37°C, unless stated otherwise.

### NGS sequence analysis and error filtering

The Complex Heterogeneous Overlapped Paired-End Reads (CHOPER) filtering approach that was developed in this study for error filtering and mutation detection comprised three steps:

- Perfect Self-Aligned Block (PSAB)
- Gapless Reference-Aligned Sub-Block (GRASB)
- Complete Codon Sub-Block (CCSB)

(i) PSAB

The forward and reverse reads were aligned to each other using JAligner (see the section on Software packages used, below). The unpaired tails were discarded (primers, etc.). The longest internal block that had no gaps and no mismatches, called the Perfect Self-Aligned Block (PSAB), was extracted from each such aligned pair.

(ii) GRASB

The PSABs were aligned to the reference sequence (here, the p53-M237I core domain) using JAligner. The longest aligned sub-block that had no gaps, called the Gapless Reference-Aligned Sub-Block (GRASB), was extracted from each aligned PSAB.

(iii) CCSB

The final filtering step produced the Complete Codon Sub-Block (CCSB). It discarded (1) any GRASB with a mismatch to the reference sequence at more than one codon position, (2) incomplete codons at the beginning or end of each GRASB, and (3) all remaining sub-blocks smaller than 12 base pairs long. Any read differing by more than one codon from the reference sequence must contain an error, because the ACS mutagenesis procedure produces only a single changed codon in each mutagenesis product. Incomplete codons at the beginning or end may be ambiguous in identifying the changed codon. The choice of sub-block length is an engineering trade-off between the wish to make the sub-block as short as possible, so that few candidate reads are lost, and the wish to make it as long as possible, so that most ambiguous alignments are avoided. Table B in S1 File and Figure A in S2 File illustrate this trade-off by showing the number of ambiguous ACS alignments (there called “collisions”) that resulted for various choices of sub-block length. As shown there, sub-blocks smaller than 12 base pairs produced many ambiguous alignments to the reference sequence, while sub-blocks longer than 12 base pairs did not appreciably reduce further the percentage of ambiguous alignments. Related applications should conduct a similar analysis and choose their sub-block length according to the data for their problem.

### Phred quality score filtering

Illumina provides a Phred quality score [22,23] for each nucleotide, which indicates the estimated probability that a given base is incorrect. The quality score is Q = −10 log_10_(P), where P is the error probability. We performed two computational experiments to compare the results of our filtering procedure with Phred quality score filtering. In the first Phred experiment, the longest blocks with a minimum quality score of 30 were extracted from each forward read. This experiment was conducted to investigate the degree to which Phred quality filtering alone can reduce sequencing errors in the specific problem addressed here. In the second Phred experiment, the quality score of 30 was applied as an optional additional filter at the beginning of our procedure to assess its effect on the final accuracy of our CHOPER filtering results. In this experiment, all base pairs in the forward and reverse reads with Phred scores smaller than 30 were marked as “N” (any base). The CHOPER filters were then applied to these reads. Alignment of an “N” to any base, including another “N”, was considered as a mismatch.

### Improved problem-specific sequence alignment algorithm

In the initial implementation of CHOPER filtering, JAligner software (http://jaligner.sourceforge.net) was used to align the Perfect Self-Aligned Block (PSAB) to the reference p53 cancer mutant M237I sequence, in order to obtain the Gapless Reference-Aligned Sub-Block (GRASB) and ultimately the Complete Codon Sub- Block (CCSB). JAligner implements the well-established Smith-Waterman local alignment method [24] with *O(mn)* time complexity, where *m* is the length of the reference sequence and *n* is the length of the read. This alignment step consumed most of the time in CHOPER filtering.

To improve the efficiency of CHOPER filtering, a novel fast alignment algorithm with *O(n)* time complexity was developed that is specialized to CHOPER. This approach takes the forward and reverse reads as input and generates the final filtered output in a single step.

This new alignment algorithm essentially follows the seed-and-extension paradigm, which can be traced back to BLAST [25,26]. Many alignment algorithms afterwards employed modified or improved seed-and-extension methods for different alignment applications [27–33]. All these methods are based on hash table indexing, which can be applied either on reads, for example in BLAST [25], or on genomes, for example in SOAP [27]. The alignment algorithm developed here is similar to those alignment approaches only in the sense of using the indexing and extension paradigm. It is specifically designed for sequence analysis problems derived from All Codon-Scanning (ACS) mutagenesis and is an improvement to the alignment method originally used in CHOPER filtering.

The alignment method developed in this study comprises the following steps:

*Initialization:*

1. *Make all possible k-mers of the reference sequence that fall on exact codon boundaries*.
  Choose the smallest value of *k* that yields no ambiguous ACS alignments (“collisions”). In this study *k* = 18, as shown in Table B in S1 File and Figure A in S2 File (related studies would conduct a similar analysis). Scan the reference sequence and extract all *k*-mers that fall on exact codon boundaries. The gene for the p53 cancer mutant M237I core domain consists of 600 base-pairs (200 codons), which yields 195 such 18-mers (as 200 - [18/3-1]) for this reference sequence.
2. *Generate all possible single-codon mutations for each k-mer in step 1*.
  Since the problem of interest is mutation detection in sequences generated from All-Codon-Scanning (ACS) mutagenesis, this step generates all possible single-codon mutations of each *k*-mer from step 1 in both forward and reverse-complement senses. In this study there are (63x6+1)x195x2 = 147,810 18-mers with at most one codon difference from the reference p53 cancer mutant M237I sequence.
3. *Organize all words generated in step 2 into an efficient lookup table for search*, *called RefHash*.
  The Python dictionary data structure was used to implement the lookup table RefHash with lookup time complexity of *O(1)*. Each word in step 2 was stored as a key in the dictionary. The location of the *k*-mer on the reference sequence, the location and identity of the mutations, and whether forward or reverse strand, were stored as dictionary values.

*Iteration:*

For each paired end read pair (Read1, Read2) do:

1. *Load a lookup table*, *called ReadHash*, *with all k-mers from Read1 that have a match in RefHash*.
  Clear ReadHash. Iterate over all *k*-mers in Read1. When a *k*-mer from Read1 yields a hit in RefHash, insert that hit into ReadHash using that *k*-mer as the key and the values from RefHash as the values.
2. *Iterate over k-mers in Read2 to find the next (or first) reverse-complement hit in ReadHash*.
  Search for the next (or first) *k*-mer in Read2 having a reverse complement sequence that is found in Readhash. Terminate when the end of Read2 is reached with no next match. When a match is found, it corresponds to an exact reverse-complement match with a *k*-mer in Read1 that begins a CCSB. Its location on the reference sequence, its mutation location and identity, and whether forward or reverse strand, are given by the values from ReadHash.
3. *Extend the exact match by successive codon boundaries to form the largest possible CCSB*.
  In this step, the exact match found on the lookup table is extended. Since All-Codon-Scanning (ACS) mutagenesis mutates only one codon, during the extension only one codon mismatch is allowed between the growing CCSB and the reference sequence. Iterate over each successive codon boundary while each successive *k*-mer has a reverse-complement match in ReadHash, thereby extending the growing CCSB. The extension is terminated when a *k*-mer fails to have a reverse-complement match in RefHash, the match is not a gapless extension of the growing CCSB, a second codon mismatch is encountered, or the end of Read2 is reached. Record the extension if it is longer than the current longest CCSB.
4. *Repeat steps 2 and 3 starting from where the previous extension stopped*.
  The next exact match will be found as explained in step 2, starting from the next index beyond the end of the previous extension. It will be extended as explained in step 3. These two steps are repeated until the end of Read2 is reached.
5. *Report the longest alignment*.
  The longest extension achieved is reported as the final CCSB for this read pair.

*Proof of O(n) time complexity in Iteration step:*

Let *n* be the sum of the lengths of Read1 and Read2. Step 1 iterates over each *k*-mer in Read1 with each iteration step having time complexity *O(1)*. Thus, Step 1 has time complexity *O(*length(Read1)). In the worst case, Step 2 finds no matches in ReadHash, and so iterates over each *k*-mer in Read2 with each iteration step having time complexity *O(1)*. If Step 2 finds a match, then Step 3 iterates over Read2 by codon boundaries, which is three times faster than the iteration in Step 2. Thus, Steps 2 and 3 have time complexity *O(*length(Read2)). Consequently, the complete Iteration step has time complexity *O(*length(Read1)+length(Read2)) = *O(n)* to generate the CCSB from Read1 and Read2.

### Software packages used

JAligner, an open source Java implementation of the Smith-Waterman algorithm [24] with Gotoh's improvement [34], was used for initial sequence alignments (http://jaligner.sourceforge.net). All other codes and scripts are implemented manually.

### Mutant nomenclature

A p53 mutant is referred to herein by giving the primary cancer mutation first in capital letters, followed by secondary mutations (if any) in lower case. For example, M237I or p53-M237I refers to the p53 cancer mutant with methionine mutated to isoleucine at codon position 237, while M237I_l137r refers to M237I with leucine also mutated to arginine at codon position 137.

## Results

### Traditional sequencing results

As a control, traditional sequencing of individual colonies was used to scan the p53 cancer mutant M237I exhaustively by ACS for single-change mutations that can restore p53 function (rescue mutations) throughout the p53 core domain. We identified two novel reactivation sites with a total of 6 novel rescue mutations: M237I_l137r, M237I_r175a, M237I_r175p, M237I_r175s, M237I_r175v, and M237I_r175t. It is notable that p53-R175H is the single most common individual p53 cancer mutant found in human tumors; it has not been reactivated by any single amino acid change in previous studies [15]. Thus, it is a site already well-known to affect p53 function. Still, it is surprising that amino acid changes in position 175 can lead both to a p53 cancer mutant (p53-R175H) that cannot be reactivated by genetic mutations, and to the genetic reactivation of another cancer mutant (p53-M237I_r175h).

### NGS results

An extensive analysis was performed on the M237I, M237I_ACS, and M237I_RESCUE sample sets, as explained in the Materials and Methods section. The ability of the sequencing protocol strategy and our CHOPER error filtering and mutation detection approach was evaluated in detecting rescue mutations of p53 cancer mutant M237I.

The CHOPER error filtering and mutation detection strategy analysis results are summarized below.

**CHOPER filters reduced error rates and provided reliable read accuracy for mutation detection.** To estimate the baseline error rates in our experiments, the M237I sample set (see Materials and Methods) was analyzed comprehensively.

**Fig 1:**
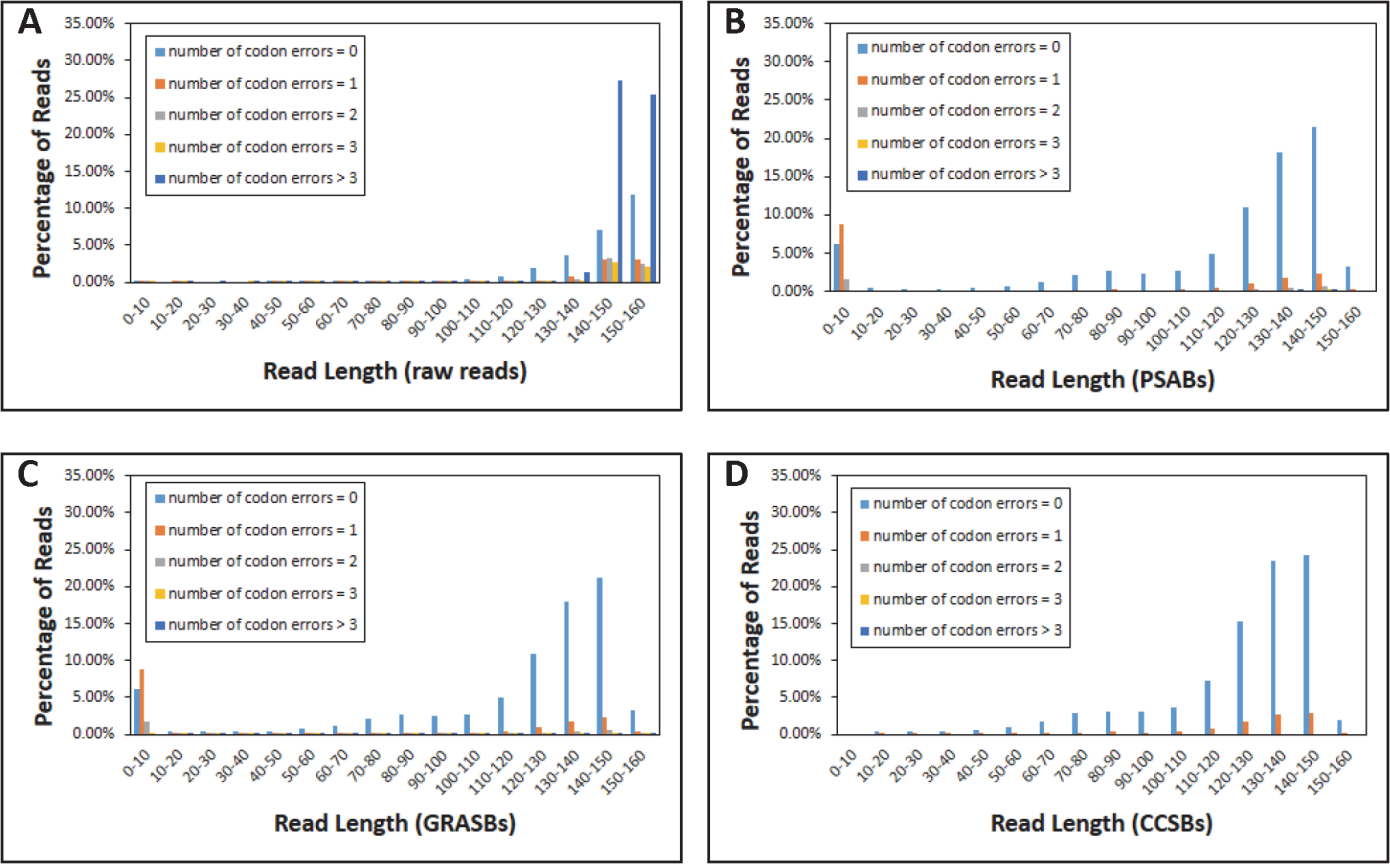
The distribution of codon errors as a function of the read lengths in M237I sample set in (A) Raw reads, (B) PSABs, (C) GRASBs, and (D) CCSBs.

**Fig 2:**
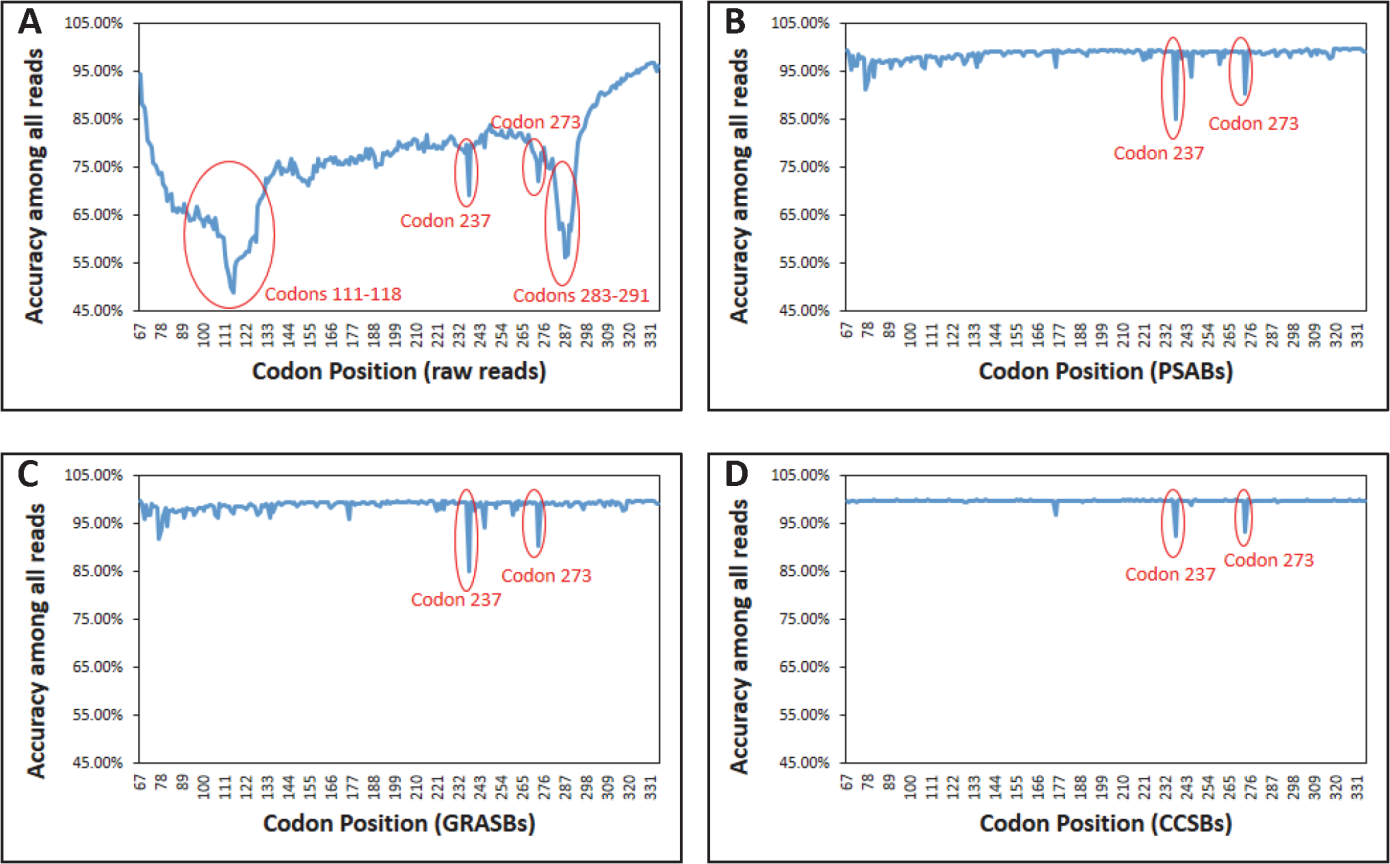
The average accuracy of reads at each codon position in M237I sample set in (A) Raw reads, (B) PSABs, (C) GRASBs, and (D) CCSBs. The regions with lower accuracy than their neighbors are circled.

First, the 20,000,000 forward reads in this sample set were aligned to the reference sequence (p53 M237I cancer mutant core domain) to compute the raw read qualities without CHOPER filtering. Figures B-A and C-A in S2 File, and Fig. 1-A show the distribution of gaps, mismatches, and codon errors, respectively, for different lengths of aligned reads. In the raw data, only 26.32% of the aligned reads were error-free (Fig. 1-A and Table 1). Fig. 2-A shows the average accuracy of the aligned reads at each codon position. The average accuracy of the aligned reads over all codon positions was 74.51% in the raw data.

**Table 1:** Analysis of M237I sample set at each step of filtering procedure.

	Raw	PSAB	GRASB	CCSB
**Total number of reads**	19,514,000	19,514,000	19,514,000	15,644,000
**Total number of base pairs**	2,453,320,000	2,039,960,000	2,032,642,000	1,931,178,000
**Average coverage of each codon position**	3,375,000	2,326,000	2,318,000	2,201,000
**Base pairs discarded**	0%	16.82%	17.14%	21.28%
**Average read length**	147	105	105	123
**Reads with no gaps**	61.03%	98.56%	100%	100%
**Reads with no Mismatches**	26.71%	78.35%	78.42%	90.10%
**Reads with no codon errors**	26.32%	77.65%	78.42%	90.10%
**Average accuracy of all codon position**	74.51%	98.65%	98.90%	99.73%

Fig. 2-A highlights four regions with lower accuracy than their neighbor regions. The low accuracies in those four regions were recovered after applying CHOPER filters (see below, also compare Fig. 2-A to 2-B, 2-C, 2-D), except specifically at codon positions 237 and 273 (see Fig. 2-B).

The exquisite sequence detail provided by NGS allowed us to determine that the low accuracy at codon positions 237 (7.5% M237 = ATG) and 273 (6.6% R273H = CAT) was most likely due to a slight p53-R273H cancer mutant contamination in the liquid culture. p53-R273H is another common cancer mutant, also under study in our laboratory. It is identical to wt p53 at codon position 237 (M237 = ATG), and also encodes the frequent human cancer mutation of arginine to histidine at codon position 273 (R273H = CAT in our laboratory version). Because positions 237 and 273 differ in location by only 36 codons (108 bp), approximately 30,000 reads spanned both positions. This number is sufficient for reliable statistical inference. In those 30,000 spanning reads, 93% of the time that we observed 273 = CAT (indicating R273H contamination) we also observed 237 = ATG (indicating reversion to wt, as in R273H); i.e., P(237 = ATG | 273 = CAT) = 0.93. Similarly, 93% of the time that we observed 273 not = CAT (indicating no R273h contamination) we also observed 237 = ATA (indicating no reversion to wt); i.e., P(237 = ATA | 273 not = CAT) = 0.93. These numbers are not expected to be exactly 1.0 because the ACS mutagenesis also would introduce some stochastic 273 = CAT mutations. Nevertheless, this single hypothesis explained almost all of the low accuracy at position 273, and explained the wt reversion at position 237 to within a residual of less than 1.5%. It is possible that this very small remaining residual is due to very slight trace contamination with wt p53, which is also under study in our laboratory; or perhaps there is a selective growth disadvantage to the M237I mutant that encourages reversion to wt. For NGS, the moral is that the detailed sequence sampling allows for fine-grained statistically-justified explanations of apparent anomalies.

Next, CHOPER filtering was applied to the M237I sample set. The 100% overlapped paired-end reads enabled us to eliminate the large majority of errors by comparing the forward and reverse reads at the very first step of our filtering process (resulting in 98.65% average accuracy over all codon positions). This significant error reduction highlighted the importance of the short fragment size selected during sequencing library preparation. For the 150 base paired-ends sequencing protocol used in this study, generation of fragments of approximately 150 bp was essential to obtain maximal complete overlap between forward and reverse reads.

The 20,000,000 forward reads were aligned to their corresponding reverse reads and the PSABs were extracted, followed by GRASBs and CCSBs (see Materials and Methods). Table 1 summarizes the analysis of M237I sample set at each filtering step. After applying all filters, the average accuracy over all codon positions increased to 99.73% at the expense of discarding 21.28% of base pairs (Table 1). Fig. 2-D shows that all low accuracy regions were recovered after applying CHOPER filters, except codon positions 237 and 273 (both explained above).

Given these encouraging results, we conducted additional experiments with the Phred quality score filter (Phred ≥ 30), both alone and added at the beginning of the CHOPER filtering pipeline (see Materials and Methods). Table C in S1 File summarizes the analysis of the M237I sample set in these experiments. First, we applied the Phred quality score filter alone, by discarding any base that failed Phred ≥ 30. The result was increased average accuracy over all codon positions from 74.51% in raw data to 86.07% with Phred filtering, at the cost of discarding 36.38% of base pairs. The accuracy was less, and the number of base pairs discarded was more, than with CHOPER filtering alone. Second, we applied the Phred quality score filter (Phred ≥ 30) as an additional optional filter before the CHOPER filters. The final accuracy after applying all CHOPER filters following Phred filtering was 99.71%, very slightly lower than the accuracy of the CHOPER filtering approach alone (99.73%, not a statistically significant difference). However, 54.47% of the base pairs were discarded when the Phred score was added as an optional filter before CHOPER, compared to only 21.28% base pairs discarded by the CHOPER filtering method alone.

The sequencing depth was reduced from 10K reads to 1M reads and the performance of CHOPER filtering was compared to Phred filtering. Figure D in S2 File shows that the average accuracy over all codon positions does not depend on the sequencing depth.

**Fig 3:**
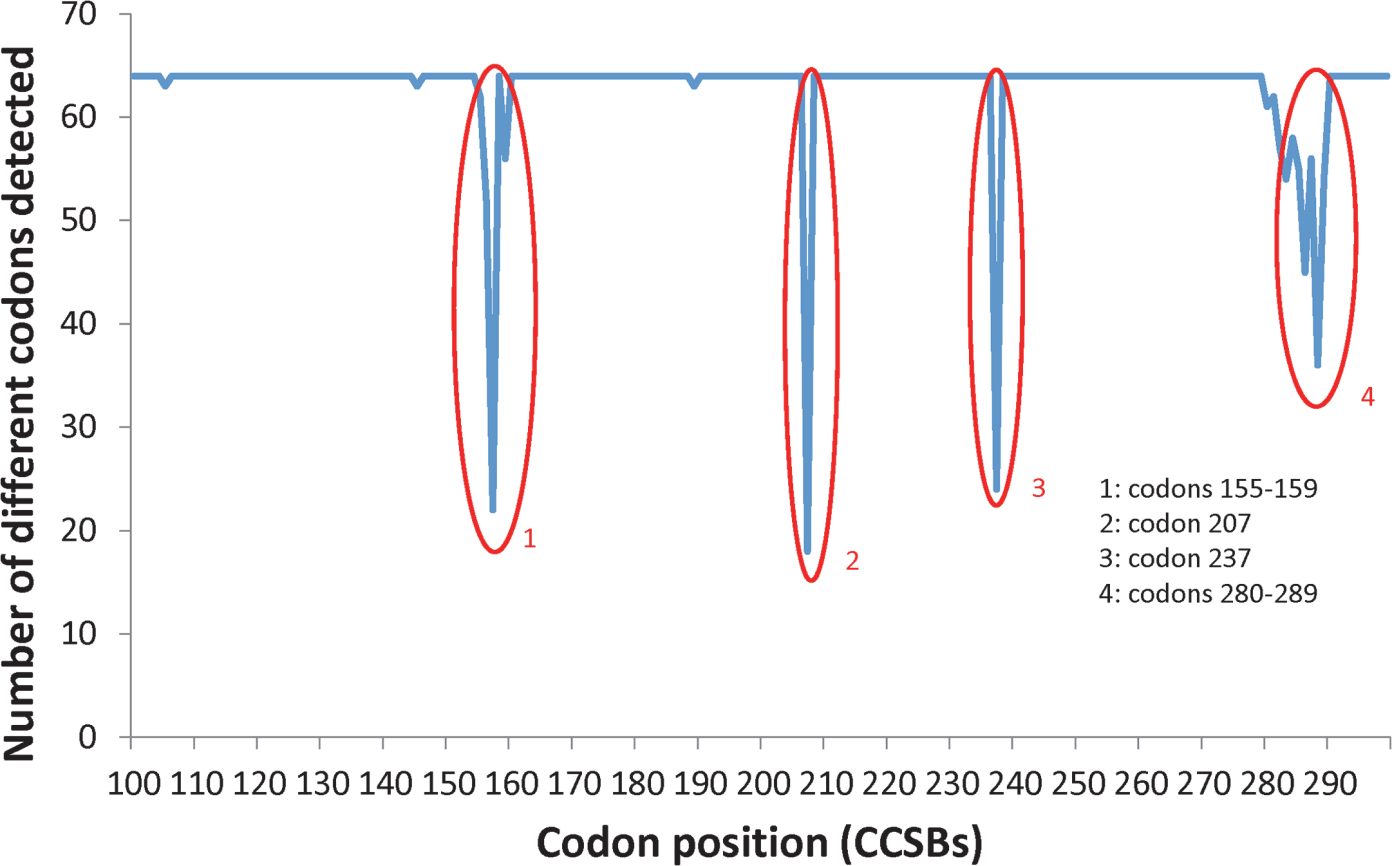
The number of different codons detected at each codon position in M237I_ACS sample set. The regions that resulted in failed in ACS reactions are circled.

**After applying CHOPER filters, 99.97% of all available codons were detected in the complex ACS library.** The M237I_ACS sample set was investigated to verify the ability of the ACS reaction to mutagenize the reference sequence to all possible codons at each position, and to confirm the ability of the CHOPER filtering approach to conserve all mutations present in the complex pool of ACS products. The ACS mutagenesis was applied at codon positions 100 to 299. Fig. 3 shows the number of different codons detected at each codon position. Figure E in S2 File shows the median number of reads for the 64 possible codons at each codon position. Fig. 3 shows that 99.97% of ACS mutations were detected in all codon positions except in four small regions (codon positions: 155–159, 207, 237, 280–289) which missed many codons. In all other codon positions (a total of 187 codon positions), the experimental procedures succeeded, resulting in 184 codon positions in which all 64 codons were observed and three codon positions in which 63 codons were observed.

We hypothesize that failure in those four small regions was due to experimental problems. If failure in mutation detection in these regions were due to a statistical or CHOPER filtering error, then we would expect to see a binomial distribution of missed codons, where the probability P of missing a codon is demonstrably a very small number based on the high coverage of all codons in almost all locations. This binomial distribution was calculated for the non-failing regions as P = m/n, where m = 3 is the number of missed codons at those 184 locations, n = 11712 is the total number of codons at those 184 locations, and P = m/n = 0.00026 is the probability of missing a given codon at any of those given 184 locations. Table D in S1 File and Figure F in S2 File show the theoretical binomial distribution of missed codons based on P compared to the experimental distribution of missed codons in the data. Table D in S1 File provides statistical evidence that the experimental data follows the theoretical binomial distribution very closely at those 184 positions. Thus, it is expected to observe that almost all positions saw all codons, a very small number of positions missed one codon, and the probability of missing two or more codons is almost zero, according to the binomial distribution with probability P. But, in fact, at codon positions 155–159, 207, 237, and 280–289 there were many missed codons. Consequently, those data cannot be explained well by the assumption of a statistical or CHOPER filter failure. Instead, those data are explained very well by an ACS mutagenesis failure. Again, for NGS, the moral is that the detailed sequence sampling allows for fine-grained statistically-justified explanations of apparent anomalies.

Since the ACS mutagenesis was designed to maintain the cancer mutant M237I, the absence of mutagenesis products at codon position 237 was expected. There are several possibilities for the minor ACS failures at a few other positions, including, but not limited to, oligonucleotide synthesis quality problems of specific primer sets, experimental errors, or deleterious cross-hybridization amongst the ACS primers at those positions. In particular, the ACS mutagenesis procedure is performed in groups of 10 codons (100–109, 110–119,…, 290–299), each using a pool of 10 mutagenesis oligonucleotides, and so each of the problem areas shown in Fig. 3 is contained within a single ACS group of 10 oligonucleotides. All other codons were successfully mutagenized by ACS, and the CHOPER filtering method observed 99.97% of all possible available codons at each such successful codon position.

Figure G in S2 File shows the average number of different codons detected at each location for different sequencing depths. The expected number of different codons detected at each location follows the Binomial Distribution. Sheared products were in the range of 130–150 bp (see Methods and Materials), and so assuming an average sheared product length of 140 bp yields an average of 46 complete codons per read. Thus, the probability *p* of observing any given codon change at any given codon location within any given read is p = (46/200)x(1/64)x(1/200) = 0.00001796875 so the probability *P* of observing any given codon change at any given codon location after *N* reads is p = 1–(1—p)^N^ and the expected average number of changed codons per position observed in *N* reads, *AvgCodons(N)*, is AvgCodons(N) = Px63. Table G in S1 File gives the expected average number of changed codons per position observed in *N* reads, for various values of N.

**CHOPER filters detected rescue mutations available in the pool of complex heterogeneous samples.** The M237I_RESCUE sample set was analyzed to validate the CHOPER filtering method in detecting the mutations that restore the activity of p53 cancer mutant M237I (“rescue mutations”). Table 2 lists rescue mutations that we identified for cancer mutant M237I using traditional sequencing of individual yeast colonies. These rescue mutations were expected to be detected if the CHOPER filtering process succeeded. The codons encoding each of these rescue mutations, together with the fold changes in the filtered reads before and after selecting for p53 activity, are also shown in Table 2. The rescue mutations were the only mutations detected in the M237I_RESCUE sample set with fold-increases greater than one after applying CHOPER filters. The CHOPER filtering method successfully removed the errors present in this sample set and thus overcame the main concern for detection of rare sequence variants. Table 2 shows that all the rescue mutations of p53 cancer mutant M237I were detected in our sample set, except for rescue mutation M237I_l137r, which was identified as a rescue mutant by traditional sequencing methods. All these available rescue mutations were detected even at sequencing depths as low as 10K reads.

**Table 2:** Rescue mutations of M237I cancer mutant.

“Cancer rescue” mutation	Rescue mutation codon	Fold increase*	Codon Usage data [35,36]
M237I_l137r	AGA, AGG	0	21.3, 9.2
	CGT, CGA, CGC, CGG	0	6.5, 3.0, 2.6, 1.7
M237I_r175a	GCC	505	12.5
	GCA	909	16.1
	GCG	363	6.1
	GCT	591	21.1
M237I_r175p	CCT	545	13.6
	CCA	483	18.2
	CCG	461	5.2
	CCC	505	6.8
M237I_r175s	TCG	50	8.5
	TCA	102	18.8
	TCC	136	14.2
	TCT	283	23.5
	AGT	2	14.2
	AGC	18	9.6
M237I_r175t	ACT	392	20.3
	ACC	524	12.6
	ACG	138	7.9
	ACA	332	17.8
M237I_r175v	GTT	600	22
	GTC	536	11.6
	GTA	442	11.8
	GTG	466	10.6

*Fold increase = (Total number of the given codon in M237I_RESCUE sample set / Total number of the given codon in M237I_ACS sample set)

We conducted further experiments to elucidate more properties of the putative M237I_I137r rescue mutant and to understand why NGS failed to detect M237I_l137r. Mutant M237I_I137r was identified by traditional sequencing as a rescue mutation based on growth on solid media, where cell colonies are physically separate from each other and growth, however slow, continues until an identifiable colony forms. However, the NGS experiment was performed in liquid growth media, in which each cell clone population must compete for nutrients with every other clone and the clone growth rate is proportional to its fitness. We therefore suspected that mutant M237I_l137r might have a competitive growth disadvantage as compared to other rescue mutations.

**Fig 4:**
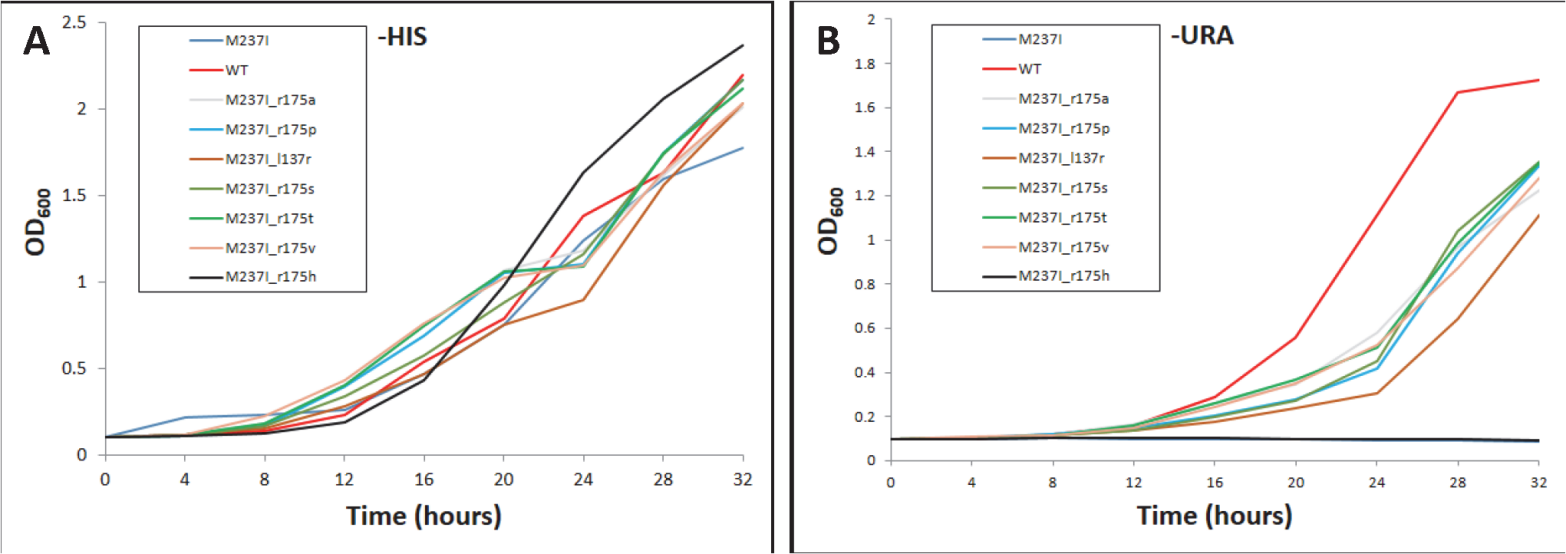
Growth curve analysis of yRB239 yeast expressing p53 wild-type, M237I cancer and rescue mutants, (A) non selective (-HIS), (B) selective (-URA) liquid medium. Absorbance by optical density analysis at 600 nm was assessed over a 32-hour time course by plate reader. Measurements represent the average of two repetitions.

Indeed, M237I_l137r grew significantly slower in liquid medium as compared to all other rescue mutants (Fig. 4). Surprisingly, changing leucine to arginine at codon position 137 seemed to introduce a dominant negative effect because growth of M237I_l137r was even inhibited when cells were grown in media supplemented with histidine (-HIS medium) where p53 activity was not required for growth. Fig. 4 shows the growth curve analysis of yRB239 yeast expressing p53 wild-type, M237I, cancer, and rescue mutants for liquid mediums either non-selective (-HIS), or selective (-URA) for p53 activity.

**Codon usage (the Codon Adaptation Index) was an important factor in strain growth rates and mutation occurrence frequencies.** Finally, we also found that for the NGS methods we employed, considering amino acid changes alone was insufficient to explain observed occurrence frequency. The effect of DNA codon usage in calculating mutant fitness based on codon occurrence frequency could not be ignored. The correlation between codon usage [35,36] and fold changes in rescue mutations of Table 2 was calculated. Table E in S1 File shows that DNA codon usage (i.e., the Codon Adaptation Index [35,36]) was well correlated with strain growth.

### Novel alignment algorithm results

The novel fast alignment algorithm explained in materials and methods section was implemented in Python and run on the M237I sample sets. The Perfect Self-Aligned Blocks (PSABs) from CHOPER filtering were used as input and the Complete Codon Sub-Block (CCSBs) were generated in a single step as output. This novel alignment method successfully generated the same high accuracy (99.73%) as the original CHOPER filtering approach. In addition, this novel alignment method discarded only 19.33% of base-pairs in the final results (compared to 21.28% in the initial CHOPER filtering implementation reported above). More importantly, the average run time of this method was ten times faster than the original implementation using JAligner. In addition, the average run time of CHOPER filtering was compared to BLAST [25]. Figure H in S2 File shows that the average run time of CHOPER filtering is ten times faster than BLAST. This alignment algorithm extends the efficiency of CHOPER filters and is a proper replacement for the Smith-Waterman algorithm in this application.

### CHOPER filtering performance on simulated reads

Simulation studies were conducted to analyze CHOPER performance on a longer genome region. Cystic fibrosis transmembrane conductance regulator (CFTR) with ∼4400 bp was used to generate simulated reads [37,38]. 1,000,000 forward and reverse reads were generated at random locations of gene. Mutations, insertions, and deletions were added at random locations of paired reads. The average accuracy of all codon positions were calculated before and after applying CHOPER filters. Table F in S1 File shows that CHOPER filters removed all random errors at all codon positions.

## Discussion

Application of NGS methods for identification of minor variants in highly complex heterogeneous samples has been difficult due to the high error rates of current NGS methods and the limited error filtering techniques that are available. Eren *et al*. [39] compared the complete overlap methodology on the Illumina platform with two recently published approaches based on quality score filtering (see methods) by Bokulich *et al*. [40] and Minoche *et al*. [41]. The Eren *et al*. study showed that, while those methods perform well in recognizing most low-quality reads, they tend to identify many reads with random sequencing errors as high-quality reads.

This paper described a two-arm study that compared traditional sequencing against NGS sequencing results to detect rare sequence variants. We addressed an important biological problem that involved rare mutations in a complex heterogeneous population, namely, discovery of cancer rescue mutations in the p53 protein, which is mutated in ∼50% of all human cancers. ACS enabled a simple approach for exhaustive library preparation of p53 cancer and rescue mutants [15]. However, sequencing using traditional methods was still very expensive and labor intensive. Identification of rescue mutants by traditional methods required colony growth on solid media. The number of colonies to sequence was determined *ad hoc*, based on initial results and probabilistic calculations.

In response to these problems, we developed a novel computational error filtering strategy for ACS mutagenesis (CHOPER) that is built upon complete overlap paired-end NGS reads. The CHOPER filtering approach introduced in this study enabled detection of all available rescue mutations of p53 cancer mutant M237I present in the library. We showed that the CHOPER filters increased the average accuracy of pruned reads 13% more than Phred quality score filtering alone, which supports the conclusions of Eren *et al*. [39]. In addition, combining Phred quality filtering with CHOPER filtering did not improve the final accuracy of pruned reads compared to CHOPER filtering alone, and also discarded twice as many bases as the CHOPER filtering approach alone.

One limitation of the NGS method as described here, using liquid media (-URA) compared to traditional sequencing using solid media (-URA), is that it might miss legitimate low-growth rescue mutants due to their unusually low growth rates. Their low growth rates could cause them to be drastically under-represented in the liquid media sample. A revised and improved NGS methodology for future experiments would be to combine (1) colony selection on solid medium, which would overcome growth disadvantage of mutants showing growth inhibitory effects unrelated to p53 activity, with (2) occurrence frequency after direct competition during growth in liquid media, which estimates rescue strength. A combination of both approaches could provide the most complete picture of overall mutant properties. In addition, NGS methods cannot ignore the effect of DNA codon usage [35,36] in calculating mutant fitness based on occurrence frequency. DNA codon usage (i.e., the Codon Adaptation Index [35,36]) was well correlated with strain growth.

The results presented here extended NGS application into yet another area involving heterogeneous genomes, an area that previously has been difficult for NGS methods.

## Supporting Information

S1 File All supporting tables and supporting figure legends. (DOCX)

S2 File All supporting figures. (ZIP)
