# Supplementary material for: CHOPER Filters Enable Rare Mutation Detection in Complex Mutagenesis Populations by Next-Generation Sequencing
Source: PLoS One. 2015 Feb 18;10(2):e0116877. doi: 10.1371/journal.pone.0116877 (PMC4333345; doi:10.1371/journal.pone.0116877)
Supplement: S1 File — (DOCX) [file pone.0116877.s001.docx]

**Supporting Information**

1. Supporting Tables

2. Supporting Figure Legends

**1. Supporting Tables**

Table A. Schematic description of the three NGS libraries.

| **Library** | **Origin** | **Expected NGS result** |
| --- | --- | --- |
| **M237I** | Native human p53 molecule with codon position 237 mutated from methionine (ATG) to isoleucine (ATA) | Reads that closely match native human p53, except that codon position 237 will be ATA instead of ATG |
| **M237I_ACS** | Library M237I after ACS mutagenesis, which results in every codon position being mutated to every possible codon, while guaranteeing at most one mutated codon in each mutagenesis product | Reads that closely match Library M237I, except that single-codon mutations will appear in proportion to their ACS mutagenesis incorporation frequency |
| **M237I_RESCUE** | Library M237I_ACS after selection for active p53 double mutants (“cancer rescue” mutants) by the yeast assays described in the text | Reads that correspond to those codon mutations in Library M237I_ACS that reactivate p53 cancer mutant M237I, with the frequency of reads from each rescued mutant proportional to their growth rate, and hence to their degree of p53 reactivation, after normalization by their mutagenesis incorporation rate in Library M237I_ACS |

Table B. The Percent Collisions for various choices of Sub-block Length. For various choices of sub-block lengths, all possible legal ACS mutations were generated as described in the manuscript for both the forward and the reverse sense. A given mutation of a given sub-block was deemed to be a “collision” if its resulting sequence was identical to that of any other previously-generated such mutation, again considering both the forward and the reverse sense. The total number of collisions was divided by the total number of sub-block mutations and expressed as a percent to compute the Percent Collisions for various choices of Sub-block Length.

| **Sub-block Length** | **Percent Collisions** |
| --- | --- |
| 3 | 99.75 |
| 6 | 91.77 |
| 9 | 18.53 |
| 12 | 0.79 |
| 15 | 0.02 |
| 18 | 0 |
| 21 | 0 |

Table C. Analysis of M237I sample set at each step of filtering procedure with an additional Phred ≥ 30 quality filtering.

|  | **Raw** | **Phred ≥ 30** | **PSAB** | **GRASB** | **CCSB** |
| --- | --- | --- | --- | --- | --- |
| **Total number of reads** | 19,514,000 | 19,330,000 | 19,308,000 | 19,308,000 | 13,579,000 |
| **Total number of base pairs** | 2,453,320,000 | 1,560,823,000 | 1,206,503,000 | 1,202,797,000 | 1,116,843,000 |
| **Average coverage of each codon position** | 3,375,000 | 1,900,000 | 1,299,000 | 1,296,000 | 1,224,000 |
| **Base pairs discarded** | 0.00% | 36.38% | 50.82% | 50.97% | 54.47% |
| **Average read length** | 147 | 87 | 62 | 62 | 82 |
| **Reads with no gaps** | 61% | 85% | 99.32% | 100% | 100% |
| **Reads with no Mismatches** | 26.71% | 58.40% | 85.09% | 85.13% | 93.36% |
| **Reads with no codon errors** | 26.32% | 58.03% | 84.72% | 85.13% | 93.36% |
| **Average accuracy of all codon position** | 74.51% | 86.07% | 98.43% | 98.60% | 99.71% |

Table D. The probability of missing codons in a binomial distribution with P=0.00026 and the observed frequency in experimental data (Table C corresponds to Fig. D).

| **Number of missed codons** | **Probability (theoretical binomial distribution)** | **Frequency (experimental data)** |
| --- | --- | --- |
| 0 | 0.983738133 | 0.983606557 |
| 1 | 0.016130987 | 0.016393443 |
| 2 | 0.000130189 | 0 |
| 3 | 6.89358E-07 | 0 |
| 4 | 2.69349E-09 | 0 |
| 5 | 8.28131E-12 | 0 |
| 6 | 2.08642E-14 | 0 |
| 7 | 4.42927E-17 | 0 |
| 8 | 8.08572E-20 | 0 |
| 9 | 1.28904E-22 | 0 |
| >=10 | 1.81876E-25 | 0 |

Table E. The correlation between fold increases of M237I_r175x rescue codons in Table 2 and codon usage frequencies in Sharp *et al.* [35,36].

| **Cancer_rescue mutation** | **Correlation (r)** |
| --- | --- |
| M237I_r175a | 0.59 |
| M237I_r175p | 0.51 |
| M237I_r175s | 0.81 |
| M237I_r175t | 0.47 |
| M237I_r175v | 0.83 |

Table F. CHOPER performance on cystic fibrosis transmembrane conductance regulator (CFTR) simulated reads. CFTR has ~4400 bps. 1,000,000 forward and reverse reads were generated at random locations of gene. Mutations, insertions, and deletions were added randomly to the reads.

|  | **Raw** | **CHOPER** |
| --- | --- | --- |
| Average read length | 152 | 58 |
| Total % of gaps | 3.92% | 0% |
| Total % of mismatches | 6.40% | 0.00% |
| Average accuracy of all codon positions | 89.68% | 100.00% |

Table G. Read Number *N* and Average Number of Observed Changed Codons *Avg(N)*.Compare to Figure G.

| **Read Number *N*** | **Average Number of Observed Changed Codons *Avg(N)*** |
| --- | --- |
| 15625 | 15.42 |
| 31250 | 27.07 |
| 62500 | 42.51 |
| 125000 | 56.33 |
| 250000 | 62.29 |
| 500000 | 62.99 |
| 1000000 | 63.00 |
| 2000000 | 63.00 |
| 4000000 | 63.00 |

**2. Supporting Figure Legends**

Figure A. The Percent Collisions for various choices of Sub-block Length.

Figure B. The distribution of gaps as a function of the read lengths in the M237I sample set in (A) Raw reads, (B) PSABs, (C) GRASBs, and (D) CCSBs.

Figure C. The distribution of mismatches as a function of the read lengths in the M237I sample set in (A) Raw reads, (B) PSABs, (C) GRASBs, and (D) CCSBs.

Figure D. The average accuracy of all codon positions at different sequencing depth using CHOPER filtering and Phred ≥ 30 filtering.

Figure E. The median number of reads for the 64 possible codons at each codon position in the M237I_ACS sample set. The regions that show failed ACS reactions are circled.

Figure F. The probability of missing codons in a binomial distribution with P=0.00026 and the observed frequency in experimental data.

Figure G. The number of different codons detected at different sequencing depths using CHOPER filters.

Figure H. The average run time of CHOPER filtering compared to BLAST for various numbers of reads.
