# Supplementary figures and images for: CHOPER Filters Enable Rare Mutation Detection in Complex Mutagenesis Populations by Next-Generation Sequencing
Source: PLoS One. 2015 Feb 18;10(2):e0116877. doi: 10.1371/journal.pone.0116877 (PMC4333345; doi:10.1371/journal.pone.0116877)

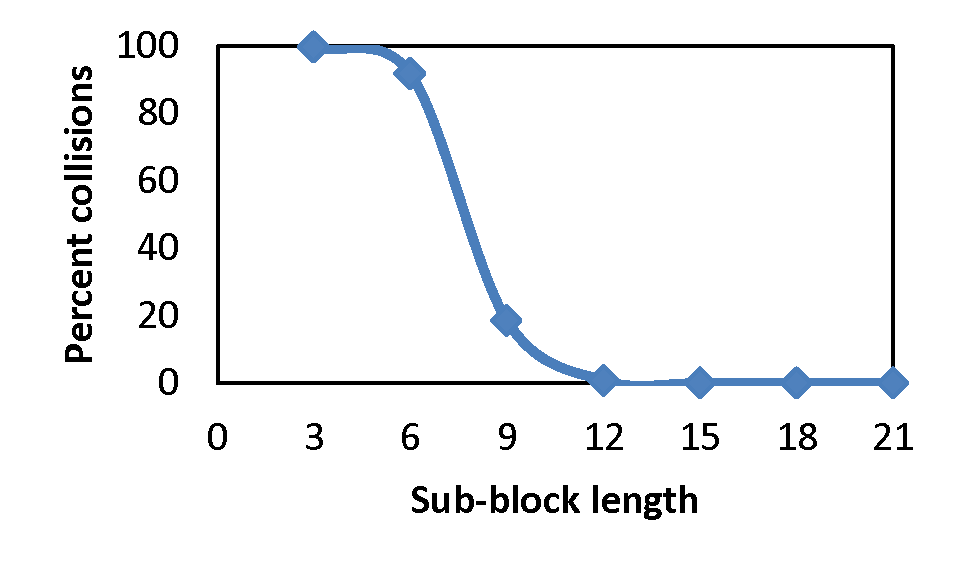

Supplement: S2 File — (ZIP) [file pone.0116877.s002.zip › S2_File/Figure A.tif]

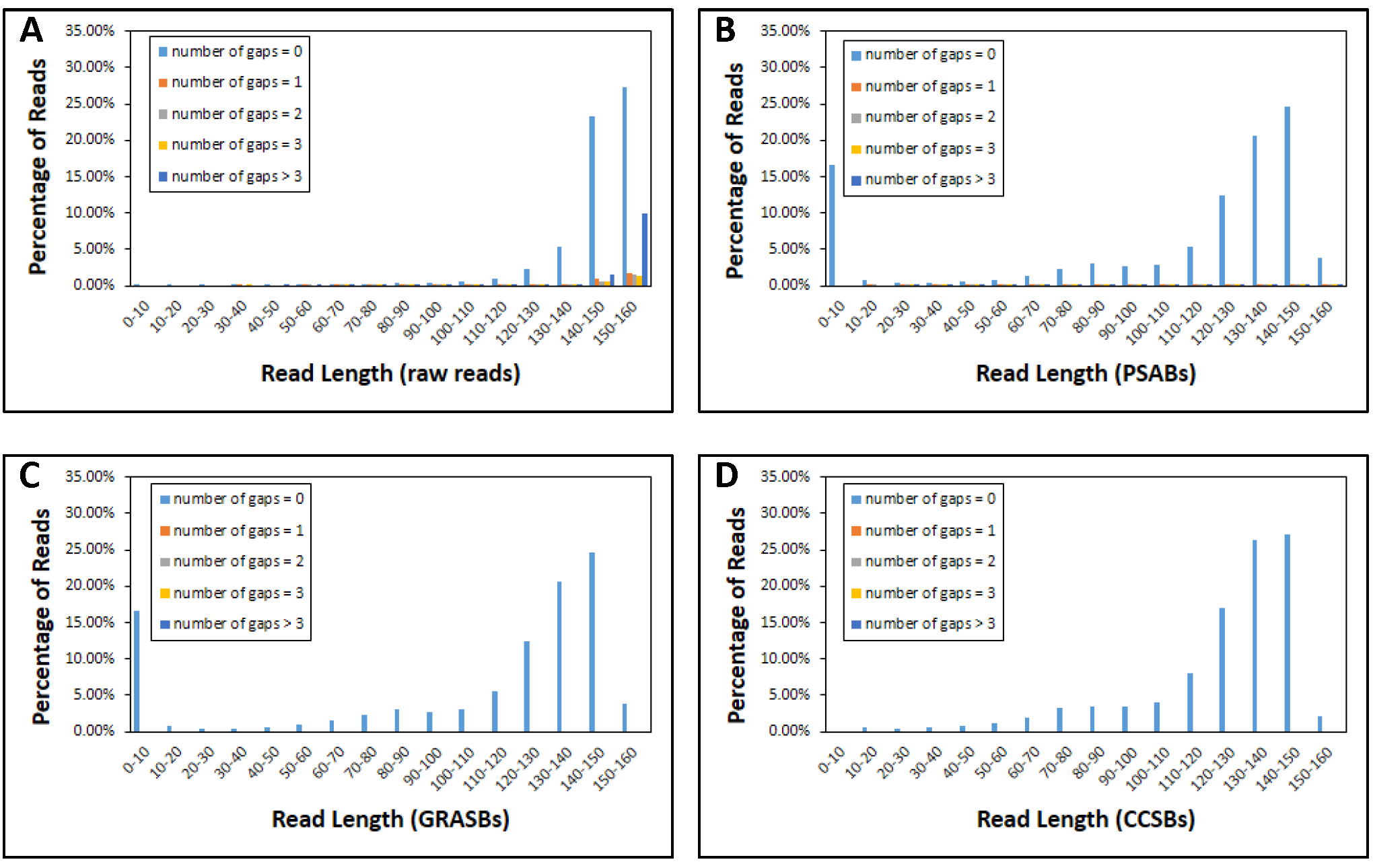

Supplement: S2 File — (ZIP) [file pone.0116877.s002.zip › S2_File/Figure B.tif]

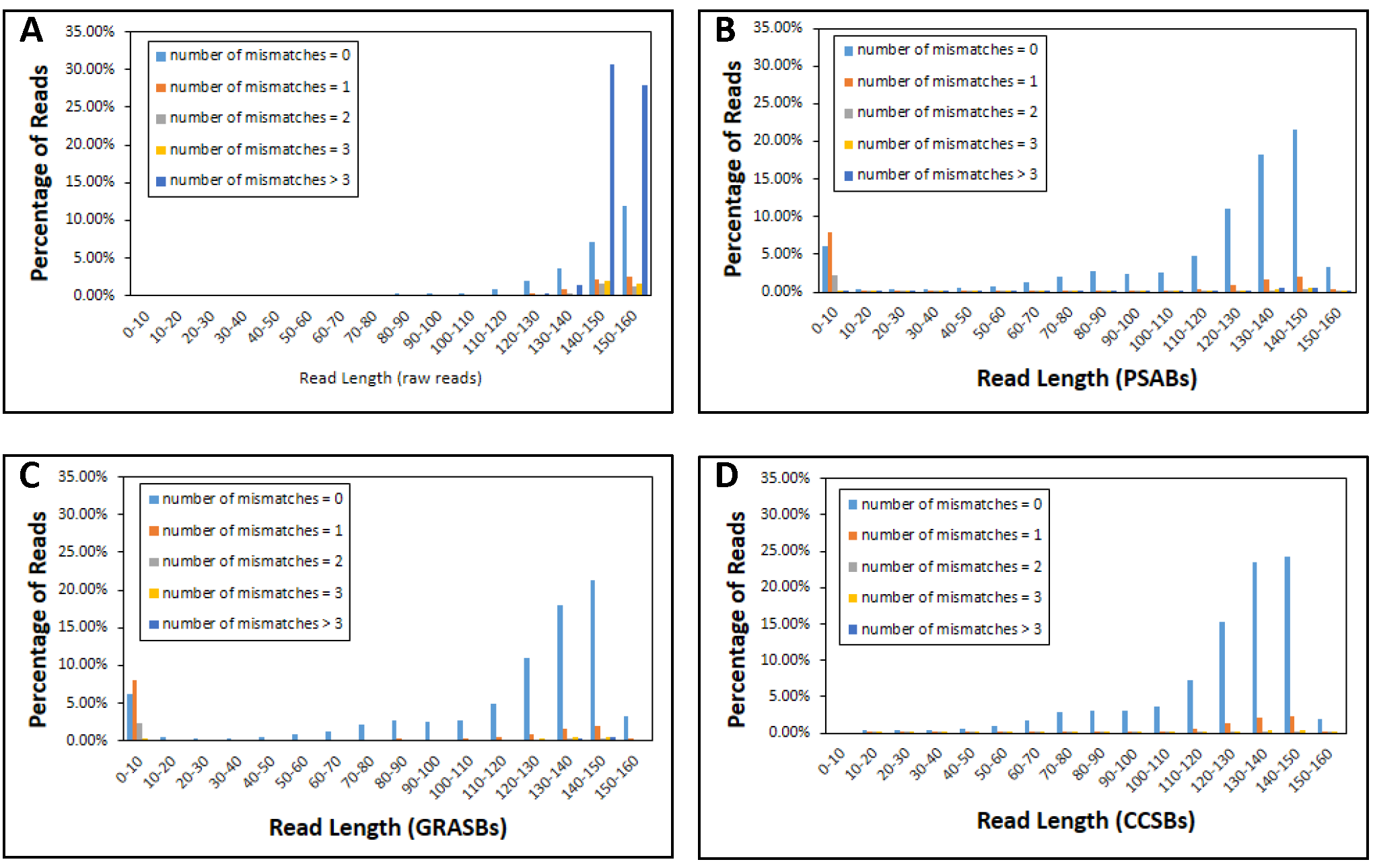

Supplement: S2 File — (ZIP) [file pone.0116877.s002.zip › S2_File/Figure C.tif]

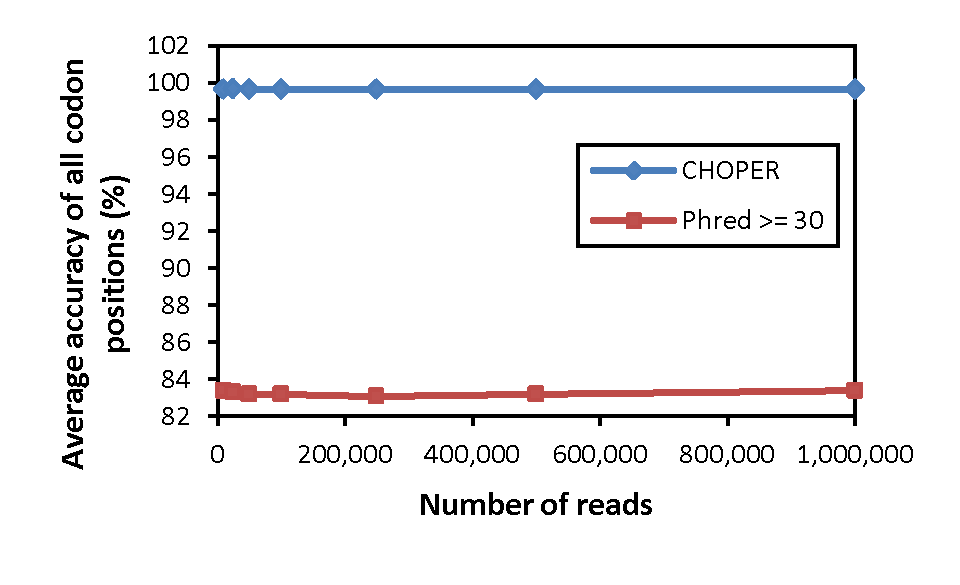

Supplement: S2 File — (ZIP) [file pone.0116877.s002.zip › S2_File/Figure D.tif]

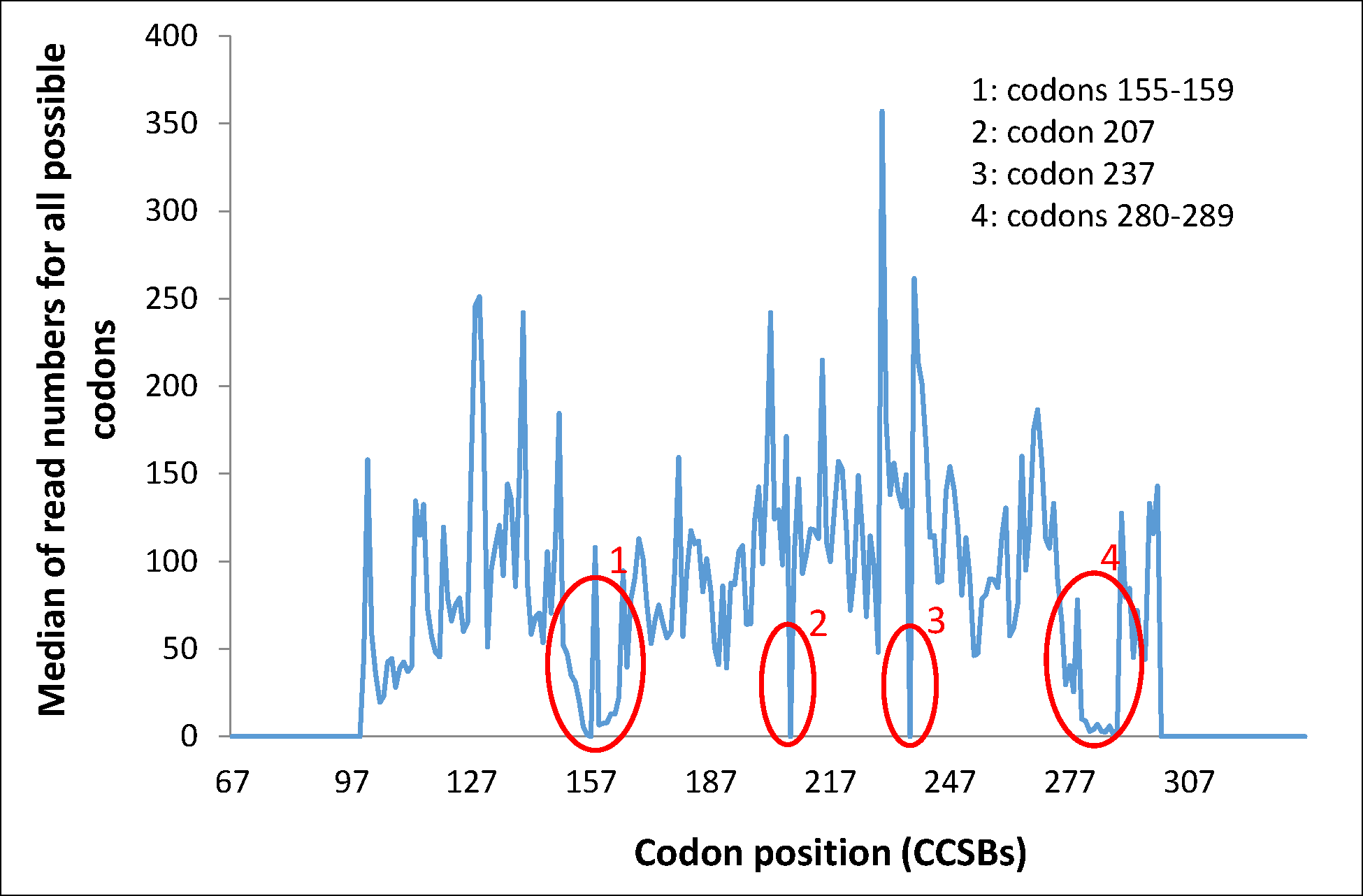

Supplement: S2 File — (ZIP) [file pone.0116877.s002.zip › S2_File/Figure E.tiff]

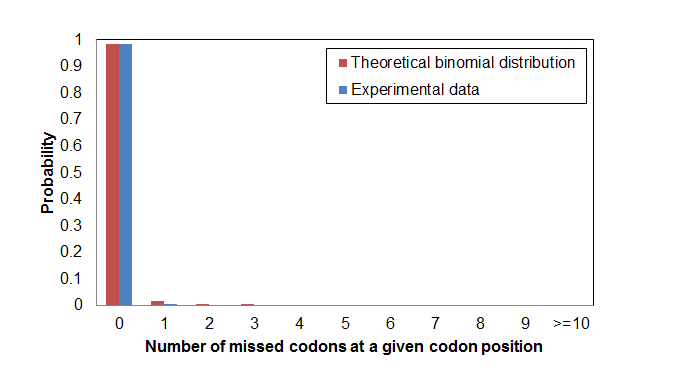

Supplement: S2 File — (ZIP) [file pone.0116877.s002.zip › S2_File/Figure F.tif]

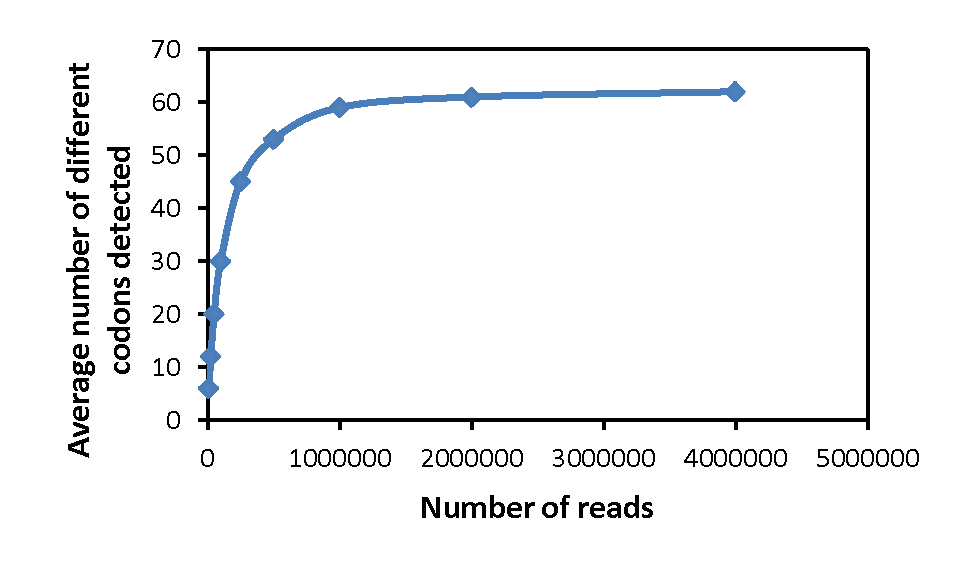

Supplement: S2 File — (ZIP) [file pone.0116877.s002.zip › S2_File/Figure G.tiff]

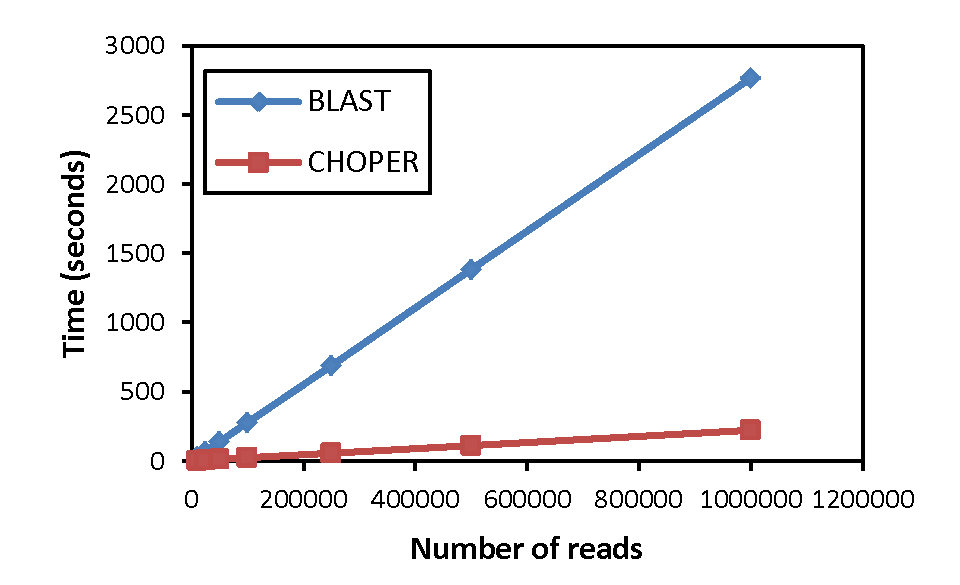

Supplement: S2 File — (ZIP) [file pone.0116877.s002.zip › S2_File/Figure H.tiff]
